# Beyond the present: current and future perspectives on the role of infections in pediatric PCD

**DOI:** 10.3389/fped.2025.1564156

**Published:** 2025-03-18

**Authors:** Megan Frohlich, Bernadette Prentice, Louisa Owens, Shafagh Waters, Lucy Morgan

**Affiliations:** ^1^ Discipline of Paediatrics and Child Health, School of Clinical Medicine, Faculty of Medicine and Health, UNSW Sydney, Sydney, NSW, Australia; ^2^ Department of Respiratory Medicine, Sydney Children’s Hospital, Sydney, NSW, Australia; ^3^ Molecular and Integrative Cystic Fibrosis Research Centre, UNSW Sydney, Sydney, NSW, Australia; ^4^ School of Biomedical Sciences, Faculty of Medicine and Health, UNSW Sydney, Sydney, NSW, Australia; ^5^ Department of Respiratory Medicine, Concord Hospital, Sydney, NSW, Australia; ^6^ Faculty of Medicine, University of Sydney, Sydney, NSW, Australia

**Keywords:** primary ciliary dyskinesia, acute infection, airway infection, exacerbation, bronchiectasis, airway remodeling, mucociliary clearance, microbiome

## Abstract

**Introduction:**

Primary Ciliary Dyskinesia (PCD) is a rare genetic disorder affecting motile cilia, leading to impaired mucociliary clearance and increased susceptibility to respiratory infections. These infections contribute to long-term complications such as bronchiectasis and lung function decline.

**Objectives:**

This review explores both the acute and long-term impact of respiratory infections in children with PCD, while highlighting the multiple contributors to infection susceptibility. The review also evaluates emerging personalized approaches such as gene and mRNA therapy that hold promise for restoring ciliary function and reducing the burden of acute infections in pediatric PCD.

**Key findings and conclusions:**

Acute respiratory infections have a significant impact on morbidity in pediatric PCD, driving progressive airway remodeling. While current treatment strategies focus on managing infections directly, emerging therapies targeting inflammation and genetic causes hold promise for reducing infection burden and improving long-term outcomes. Future advances in personalized medicine could further enhance therapeutic approaches in this population.

## 1 Introduction

Primary Ciliary Dyskinesia (PCD) is a rare, incurable, progressive genetic disorder that affects ciliary structure and function, and results in impaired mucociliary clearance (1, 2). The most common symptoms of this mucostasis are daily wet cough, recurrent respiratory tract infections, chronic rhinosinusitis and recurrent otitis media (3).

Acute infections have a substantial impact on children with PCD causing significant morbidity (4). Every exacerbation has the potential to cause a permanent decline in lung function (5) and hasten the progression of bronchiectasis (6). This review will explore the epidemiology, risks, complications, management and prevention of acute infections in children with PCD, including an exploration of the role of the microbiome in acute infections and novel personalized approaches that hold promise for restoring ciliary function.

## 2 Acute infections in PCD

**Figure 1:**
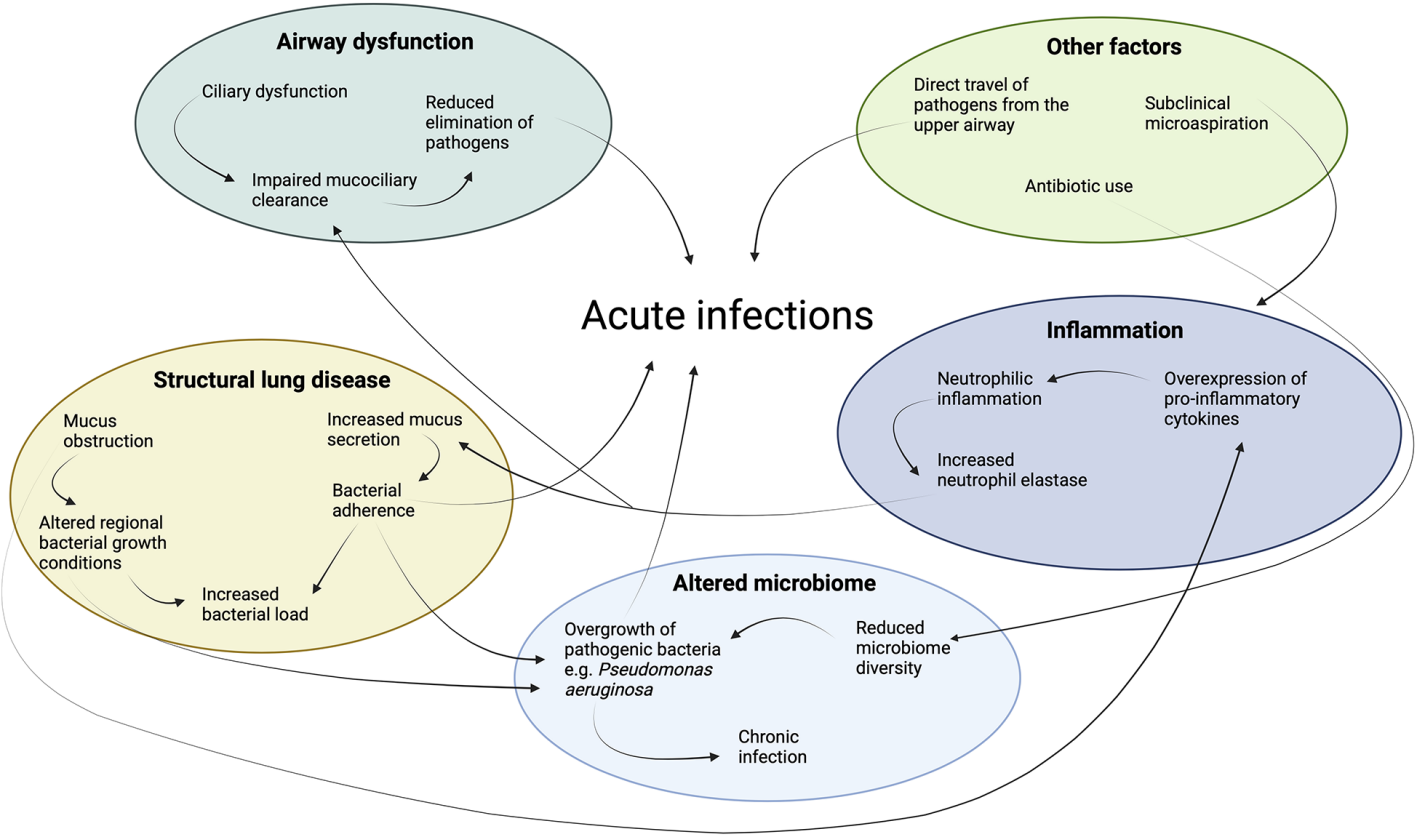
Key factors contributing to the high burden of acute infections in children with Primary Ciliary Dyskinesia (PCD). Airway dysfunction leads to impaired mucociliary clearance, promoting microbial colonization. Structural lung disease, such as bronchiectasis, exacerbates infection risk by creating areas of localized mucus obstruction and bacterial persistence. An altered microbiome in the respiratory tract contributes to the imbalance of pathogens, fostering chronic infection. Persistent inflammation due to infection and the inflammatory response causes further lung remodeling and impairment of host defences such as mucociliary clearance.

Children with PCD suffer from recurrent acute and chronic upper and lower respiratory tract infections (7). In the upper airway, the ears and sinuses are the most common sites of acute infection (3). In the lower airway, acute bacterial or viral infections are the major cause of respiratory exacerbations, with symptoms such as increased cough, change in sputum volume and/or colour, shortness of breath or fevers (8). A respiratory exacerbation is not always associated with an acute infection (9). Distinguishing an infective from a non-infective exacerbation can be challenging however due to their similar clinical presentation. Therefore, for the purposes of this review, both acute infections and exacerbations are discussed simultaneously, acknowledging that they often overlap yet remain distinct clinical events. A complex interplay of factors contribute to the high burden of acute infections in children with PCD (Figure 1).

## 3 Epidemiology of acute infections in children with PCD

Acute respiratory tract infections are common in childhood but have a more significant burden in children with PCD. Historically, collection of accurate epidemiological data on exacerbation frequency was challenging in PCD due to differing definitions in clinical practice and research. However, a consensus definition for lower respiratory exacerbations in children and adults with PCD is now available. It defines a pulmonary exacerbation as the presence of 3 or more of the following: increased cough; change in sputum colour and/or volume; decision to start or change antibiotic treatment because of perceived pulmonary symptoms; increased shortness of breath perceived by the patient or parent; new or increased hemoptysis; temperature >38 °C; and malaise, tiredness, fatigue or lethargy (8). A large international prospective cohort study was the first to collect data using this definition, reporting an incidence rate of 3.1 respiratory exacerbations per person per year in patients with PCD (10). The number of exacerbations increases with age, disease severity as measured by lung function and CT chest scores, and chronic infection with *Pseudomonas aeruginosa* (10, 11). Consensus definitions for sinonasal and otologic exacerbations are also available (12), which will facilitate collection of accurate data on these additional clinically important variables in patients with PCD.

## 4 Bacterial and viral causes of infections

Respiratory viruses are the most common cause of an acute exacerbation. In children with bronchiectasis respiratory viruses, most commonly rhinovirus, parainfluenza, influenza and coronavirus, are detected in up to 48% of exacerbations (13). The most common causative organisms for bacterial respiratory infections in PCD are *Haemophilus influenzae*, *P.aeruginosa*, *Streptococcus pneumoniae*, *Moraxella catarrhalis* and *Staphylococcus aureus* (6, 7, 14, 15). *H.influenzae* is a particularly important pathogen in children and adolescents with PCD as a cause for both acute and chronic infections, with several studies reporting a prevalence of up to 80% in this group (6, 7). The prevalence declines with age and is 21% in adults (7). Respiratory viruses can cause direct damage to the ciliated epithelial cells (16), and bacterial infections such as *P.aeruginosa* can slow ciliary beat frequency (17). This further impairs mucociliary clearance predisposing to secondary infections.

## 5 Risks and complications of acute infections in children with PCD

Acute exacerbations can cause a reduction in lung function, and approximately 25% of children with PCD fail to recover back to baseline lung function within 3 months (5). Although the overall rate of FEV1 decline in PCD is approximately 0.49 FEV1% predicted per year (18), this accelerates with a decline of 1.95 FEV1% predicted per hospitalization, based on evidence in pediatric bronchiectasis (19). Hemoptysis can occur due to acute or chronic infection and inflammation, and can be life-threatening due to rupture of a tortuous blood vessel, requiring embolization (20).

Acute infections also impact psychological wellbeing. Frequent hospitalizations have a profound impact on quality of life, leading to social isolation, missed school days, and psychological distress (4). Caregivers also experience significant mental health challenges, with acute exacerbations causing a significant worsening in quality of life and parental mental health scores (4). Strategies that reduce the frequency and severity of exacerbations have the potential to reduce the impact of having a child with PCD on the wider family.

## 6 Airway remodeling and bronchiectasis in PCD

**Figure 2:**
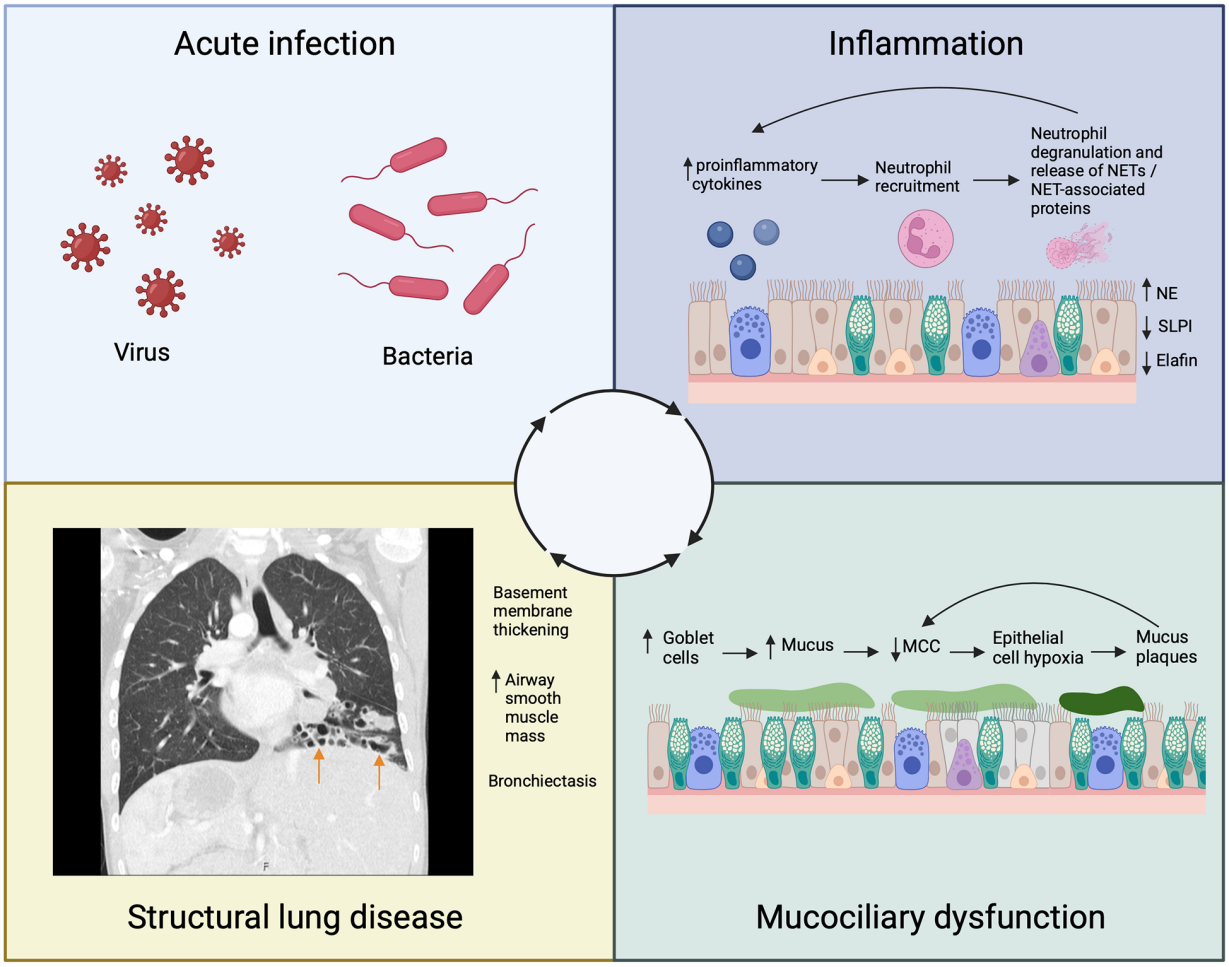
Schematic representing the contribution of acute infection to the inflammatory cycle that leads to airway remodeling and bronchiectasis (arrows) in Primary Ciliary Dyskinesia (PCD). NSPs, neutrophil serine proteases; NETs, neutrophil extracellular traps; SLPI, secretory leukocyte protease inhibitor; NE, neutrophil elastase; MCC, mucociliary clearance.

Acute infections contribute to structural changes in the airway walls by initiating and propagating the “vicious vortex” cycle of infection, inflammation, mucociliary dysfunction and structural lung disease (Figure 2) (21). Understanding this vortex is vital in bronchiectasis, whatever the underlying cause.

During an acute exacerbation, there is a significant rise in proinflammatory cytokines that attract neutrophils to the PCD airway (22). Neutrophils phagocytose pathogens and degranulate, releasing neutrophil extracellular traps (NETs) and NET-associated proteins including neutrophil elastase (NE) (23). Neutrophil elastase is a serine protease that induces goblet cell metaplasia, stimulates excess mucus production, and further impairs mucociliary clearance (24–26). The ensuing obstruction of the airway with mucus causes local epithelial hypoxia, perpetuating further pro-inflammatory events promoting neutrophil recruitment (27). Neutrophil elastase additionally impairs antimicrobial and anti-inflammatory responses by cleaving proteins such as elafin and secretory leukocyte protease inhibitor (SLPI) (24). These events further amplify the inflammatory response. In addition to driving inflammation, chronic epithelial hypoxia causes hyperconcentration of mucus due to increased transepithelial sodium and fluid absorption (28, 29). As mucus concentration increases, mucociliary transport rates further decline due to formation of mucus plaques which themselves impair ciliary function particularly in more distal airways, perpetuating further obstruction and chronic hypoxia (27).

Our understanding of the consequences of neutrophilic inflammation on airway remodeling in PCD is evolving. Bronchial epithelial reticular basement membrane thickening is seen in children with PCD on endobronchial biopsies (30, 31), and this thickening correlates with both ventilation heterogeneity measured by LCI_2.5_ and the concentration of neutrophils in lavage fluid (30). An increase in the number of myocytes and airway smooth muscle volume is also seen in children with PCD (32). Bronchiectasis is a key feature of airway remodeling in PCD, with a prevalence of 61% in children and 98% in adults (6). The degree of bronchiectasis correlates with proinflammatory cytokine and NE levels in the sputum of children with PCD (33). These findings suggest that airway remodeling may be related to the degree of neutrophilic inflammation, and the structural changes have a functional impact. As airway remodeling progresses, it likely contributes to the long-term decline in lung function observed in children with PCD emphasising the need for further research to understand the relationship between airway inflammation, remodeling and disease progression.

## 7 Role of the lung microbiome in acute infections

The human microbiome plays a crucial role in the development and progression of acute airway infections. A diverse and balanced lung microbiome helps protect the airway from infection. Beneficial microbes such as *Prevotella* and *Veillonella* limit the ability of harmful pathogens to colonise the lungs. Disruption of this balance, termed dysbiosis, allows pathogenic organisms like *H.influenzae, P.aeruginosa* or *S.pneumoniae* to cause infection (34). The lung microbiome additionally modulates the host's immune response, protecting against infection (35). Pathogen-pathogen interactions within the microbiome may also impact the course of infections. Viral infections such as influenza or rhinovirus can disrupt the upper airway microbiome, creating an environment favourable for bacterial entry into the lower respiratory tract (36). Each acute infection further alters the microbiome, creating a self-amplifying cycle of inflammation, infection, and microbiome disturbance.

Several factors contribute to alterations in the balance and composition of the lung microbiome in PCD. These include direct travel of pathogens along the airway mucosa from the upper airway, impaired mucociliary clearance, structural lung disease impacting regional bacterial growth conditions and frequent or long-term use of antibiotics (37). Many of these factors change over time, highlighting the dynamic nature of the PCD microbiome (38).

Our understanding of the PCD microbiome is evolving. Rogers *et al*. (39) used culture-independent methods to assess the lower airway microbiota of 24 adults and children with PCD using sputum and/or broncho-alveolar lavage samples. Members of the genera *Pseudomonas*, *Streptococcus* and *Haemophilus* were most abundant (39), which is in contrast to the microbiota of healthy individuals where *Streptococcus*, *Veillonella* and *Prevotella* are most abundant (40). Bacterial load was not found to correlate with lung function, however the abundance of *P.aeruginosa* was strongly negatively associated with lung function. Several other studies have linked chronic *P.aeruginosa* colonisation and more severe disease measured by lung function (41) and CT scores (11, 41) in adults and children with PCD. However, other studies have found no such association (11, 15, 42), highlighting the need for more research in this area. Tural *et al*. (43) went on to assess the composition and structure of the airway microbiota in 7 sibling pairs of children with a diagnosis of PCD and 9 healthy parents. In contrast to Rogers *et al.* (39), the most common genera were *Haemophilus*, *Actinobacillus* and *Veillonella* in patients with PCD, and *Ralstonia*, *Moraxella* and *Haemophilus* in their parents (43). Significantly less bacterial diversity was seen in patients who had a positive clinical bacterial airway culture or *H.influenzae* growth in the previous year. The microbial community tended to cluster by several clinical factors including age, lung function indices, pulmonary exacerbation status and the presence of *H.influenzae* growth. Interestingly, microbial communities did not cluster by families. These studies provide early insight into the complexity of the airway microbiota in PCD and highlight the importance of ongoing work to better understand the relationship between the microbiome and susceptibility to acute infections in PCD.

## 8 Management and prevention of acute infections

Current management and prevention strategies for acute infections in children with PCD are largely based on expert opinion and extrapolation of evidence and guidelines from other causes of bronchiectasis. Consensus recommendations for management of PCD are available from North America (1) and the European Respiratory Society (9), as well as an international consensus for infection prevention and control (44). Management of acute infections generally includes antibiotics and airway clearance techniques (ACTs). Commonly used preventative strategies include vaccinations, maintenance antibiotics, ACTs, maintenance of nutrition and exercise.

### 8.1 Antibiotic therapy

Current consensus suggests that treatment of acute exacerbations with a prolonged course of antibiotics for 14–21 days is required (45–48), but the data upon which this guidance is based are limited. Antibiotic choice is guided by the pathogens isolated from the patient's sputum or oropharyngeal cough swabs, clinical severity, local antibiotic susceptibility patterns and patient tolerance (1, 46). In the case of a severe infection or failure to respond to oral therapy, intravenous antibiotics, monitoring and intensive ACTs may be required (9, 46). If *P.aeruginosa* is isolated on lower airway samples, eradication is usually attempted (44). While there are no established *P.aeruginosa* eradication guidelines in PCD, international pediatric bronchiectasis guidelines suggest the use of IV antibiotics for 2 weeks followed by inhaled antibiotics for 4–12 weeks, if symptomatic. If asymptomatic, treatment with oral and/or inhaled antibiotics for 2 weeks followed by inhaled antibiotics for 4–12 weeks is suggested (49). The choice of antibiotics depends on local availability and preference, and *P.aeruginosa* susceptibility profiles (49). However numerous alternative eradication strategies are published and used worldwide in PCD (50–52), highlighting the need for further research to determine the optimal regime.

### 8.2 Airway clearance techniques

Airway clearance techniques commonly employed for both prevention and management of exacerbations in children with PCD include breathing techniques, manual techniques such as percussions and vibrations, positioning, positive expiratory pressure (PEP) adjuncts and oscillating devices (53). Aerobic exercise can also form an important airway clearance modality (54, 55). Studies comparing techniques have found no significant difference in lung function, acute exacerbation rate or time to first exacerbation with different methods (56–58). Therefore review by a specialist respiratory physiotherapist should be performed to decide on the most appropriate technique for each patient, taking into account factors such as their age, preference and level of experience.

### 8.3 Nebulized therapies

Hypertonic saline is the most commonly used nebulized agent in children with PCD as an adjunct to ACTs (59). Nebulized hypertonic saline osmotically hydrates airway secretions and stimulates cough (60). In a study comparing nebulized 7% hypertonic saline with 0.9% isotonic saline in 22 patients with PCD, no differences in quality-of-life outcomes was found between the two groups (61). This study had several limitations including the small sample size and lack of disease specific outcome measures. In children with non-CF bronchiectasis, 3% hypertonic saline with ACTs improves lung function parameters and reduces the number of exacerbations, compared with ACTs alone (62).

There is currently insufficient evidence to support the routine use of nebulized mucolytics such as dornase alpha in children with PCD. In adults with bronchiectasis, one large randomized controlled trial found that treatment with nebulized dornase alpha was associated with an increase in frequency of respiratory exacerbations and a greater rate of lung function decline compared with placebo, suggesting potentially harmful effects in this population (63). Despite subsequent recommendations against the use of dornase alpha in PCD (9, 48), there are several published case studies reporting improvement in lung function and symptoms in children with PCD when treated with dornase alpha, suggesting possible benefit (64–66).

### 8.4 Maintenance antibiotics

Long-term macrolide antibiotic therapy is widely used to reduce exacerbation frequency in PCD due to its anti-inflammatory, bacteriostatic and immunomodulatory properties (67). Azithromycin is the most commonly prescribed macrolide for this purpose in children, but roxithromycin, erythromycin and clarithromycin have also been studied (68). A randomized controlled trial comparing maintenance azithromycin with placebo in adults and children with PCD found that azithromycin halved the rate of respiratory exacerbations (69). *In vitro,* azithromycin has been shown to inhibit production of cytokines and promote cell proliferation in airway basal cells isolated from patients with PCD (70).

## 9 Emerging preventative therapies

Ultimately, preventing acute exacerbations from occurring is the most effective long-term strategy to preserve lung function and improve outcomes in pediatric PCD. Emerging therapies such as sodium channel blockers, oral mucolytics, dipeptidyl peptidase (DPP)-1 inhibitors and gene or mRNA therapies are innovative approaches aimed at enhancing airway hydration, reducing inflammation and ultimately restoring mucociliary clearance. As research progresses, these preventative strategies hold the potential to transform PCD care, shifting the focus from reactive treatment to proactive disease modification.

### 9.1 Sodium channel blockers

A potential explanation for the apparent reduced efficacy of nebulized hypertonic saline in patients with PCD compared to those with CF is the presence of a functional cystic fibrosis transmembrane conductance regulator (CFTR) chloride channel in the PCD airway. In patients with CF, the lack of a functional CFTR channel allows the sodium-chloride (NaCl) deposited into the airway through nebulization to remain present, osmotically attracting water to hydrate mucus (71). In contrast, in PCD, the presence of a functional CFTR channel could facilitate rapid absorption of the NaCl, reducing its osmotic hydrating effect. Blocking epithelial sodium channels could theoretically slow sodium and thereby chloride and fluid absorption, improving airway hydration. This theory was investigated in CLEAN-PCD, a randomized controlled trial assessing the use of the sodium channel blocker idrevloride in patients with PCD (72). Treatment with idrevloride in hypertonic saline for 28 days led to a mean absolute change from baseline FEV1 of 1% predicted, compared with hypertonic saline alone. In the second part of the study, coadministration of the CFTR modulator therapy ivacaftor with idrevloride in hypertonic saline was found to result in additional improvement in FEV1 of 4.7% predicted from baseline. No improvement in FEV1 was seen in those treated with ivacaftor and nebulized placebo. Therefore, it was suggested by the authors that the increase in lung function seen in the ivacaftor with idrevloride in hypertonic saline group was due to the effect of prolonged treatment with idrevloride in hypertonic saline, rather than due to ivacaftor. These results highlight the potential of combining sodium channel blockers with hypertonic saline as a novel approach to enhance airway hydration, improve mucociliary clearance and potentially reduce exacerbation frequency in PCD.

### 9.2 Oral mucolytics

Oral mucolytics are an additional potential strategy to assist with preventing acute infections and reducing pulmonary exacerbations. The evidence for benefit is currently low in children with PCD. In a small placebo controlled trial of oral N-acetylcysteine (NAC), no difference in outcomes including clinical symptoms, lung function and sputum bacteriology was detected (73). REPEAT is a randomized controlled trial evaluating exacerbation rate following treatment of 104 adults and child with PCD with either azithromycin plus placebo, or azithromycin plus the oral mucolytic erdosteine (74). This study is complete and the results are in press.

### 9.3 DPP-1 inhibitors

Dipeptidyl peptidase-1 inhibitors are a particularly promising new developing therapy to prevent exacerbations in PCD, targeting neutrophilic inflammation. Neutrophil serine proteases including NE are cleaved and activated by DPP-1 during maturation of neutrophils in the bone marrow (75). DPP-1 inhibitors therefore prevent the activation of NSPs. In the phase 2 WILLOW trial, the DPP-1 inhibitor brensocatib prolonged time to first exacerbation and reduced sputum neutrophil elastase activity in adults with bronchiectasis (76). A phase 3 global study, ASPEN, has since completed enrolment and results are expected in early 2025 (77). A second DPP-1 inhibitor, BI 1291583, is being studied in the phase 2 Airleaf trial which has also finished recruitment with results pending publication (78).

### 9.4 Personalized gene and mRNA therapies

Ultimately, restoration of ciliary function and mucociliary clearance using personalized treatment approaches such as gene or messenger RNA (mRNA) therapy would be the ideal strategy to reduce the burden of acute infections in children with PCD. Gene therapy can successfully restore ciliary beat frequency (CBF) in PCD cells *in vitro*, such as those with DNAI1 deficiency, using lenti-virus-mediated delivery of complementary DNA (cDNA) (79, 80). Likewise, gene editing using tools like transcription activator-like effector nucleases (TALENs) has shown promise in correcting PCD gene defects (81). mRNA therapy, which delivers corrected mRNA encoding for a target protein to airway cells to restore protein function, has also shown great potential in PCD animal models (82, 83). Nebulized mRNA therapies targeting DNAI1 (83) and CCDC40 (84) have been developed, with promising results in the preclinical trials.

While gene and mRNA therapies hold great promise for restoring ciliary function in patients with PCD, several barriers and challenges exist. These include potential difficulties with immune related inhibition of gene or mRNA transfer (2, 85), the potential for formation of anti-drug antibodies (86–88), the unknown effect of the limited half-life of ciliated epithelial cells (89), and the genetic heterogeneity of PCD necessitating separate formulations to target each genetic defect (90). Ultimately, the successful application of gene therapy and mRNA therapies could revolutionize the management of PCD in the future.

## 10 Future directions: patient-derived airway models for studying infection and personalized therapies in PCD

The development of patient-derived airway epithelial cell models offers a transformative tool for advancing both our understanding of the infection burden in PCD and the discovery of personalized therapeutic interventions. These models, derived from nasal or bronchial epithelial cells of PCD patients, are cultured at the air-liquid interface (ALI), closely mimicking the *in vivo* respiratory environment (91). Importantly, they retain the genetic and functional characteristics of the donor (92, 93), making them invaluable for studying disease-specific pathophysiology and testing personalized drug responses. Ultimately, patient-derived airway models hold great promise for personalized drug discovery in PCD. They allow for the screening of therapeutic interventions, including antibiotics, airway clearance techniques, and novel pharmacological agents, in a patient-specific manner (92). This approach not only accelerates the development of effective treatments but also ensures that therapies are tailored to the genetic and functional characteristics of each patient, paving the way for more individualized and effective prevention and management of acute infections in children with PCD.

## 11 Conclusion

An intact mucociliary escalator is vital to protect the airway against infection. In children with PCD, acute infection has traditionally been treated as a cause of exacerbation, but it now seems clearer that both acute and chronic infection are potent drivers of chronic inflammation, precipitating and perpetuating the “vicious vortex”. The future of PCD management will require us to understand this interplay more carefully, but will also open the door to exciting opportunities to make meaningful change to the landscape for patients with PCD.
